# Supplementary material for: Irisin in Reproduction: Its Roles and Therapeutic Potential in Male and Female Fertility Disorders
Source: Biomolecules. 2024 Sep 27;14(10):1222. doi: 10.3390/biom14101222 (PMC11505643; doi:10.3390/biom14101222)
Supplement: Supplementary file 1 [file biomolecules-14-01222-s001.zip › biomolecules-3043173-supplementary.pdf]

biomolecules

MDPI

Supplementary Tables:

Table S1. Effects of Irisin on Endometrium

Effect	Description	References
**Implantation Receptiveness**	Irisin improves the receptiveness of the endometrium to blastocyst implantation, countering the effects of Mifepristone, which decreases the levels of integrin αvβ3 and LIF, markers of receptivity. Irisin treatment results in higher implantation rates compared to Mifepristone-treated groups.	[17]
**Anti-inflammatory and Antioxidative Role**	Irisin has an anti-inflammatory and antioxidative effect on uterine tissue damaged by lipopolysaccharide (LPS). It reverses LPS-induced increases in inflammatory mediators and oxidative stress markers. The beneficial effects are mediated through the AMPK-NF-κB pathway but blocked by AMPK inhibitors.	[109]

Table S2. Details on Different aspects of Preterm birth and their association with irisin

Aspect	Details
**Definition of Preterm Birth**	Birth of a newborn before completion of 37 weeks of gestation, often leading to higher risks of infant morbidity and mortality.
**Relevance of Irisin**	Irisin, derived from the FNDC5 gene, is a hormone associated with several physiological processes. Its role in pregnancy outcomes is under investigation.
**Gene of Interest**	FNDC5 gene, precursor to irisin. Variants of this gene, especially polymorphisms like rs726344 and rs1746661, are studied for their impact on preterm birth.
**Key Polymorphisms**	rs726344 G and rs1746661 A, analyzed using RFLP. These specific alleles are of interest due to their potential impact on pregnancy duration.
**Recent Findings in Women**	Women with genotype rs726344 GG show approximately 2.18-fold increased risk of preterm birth. This association has been consistently observed in diverse populations in recent studies.
**Recent Findings in Neonates**	Neonates with the rs726344 G allele exhibit about a 2.28-fold increased risk of preterm birth. Newer studies might explore additional outcomes related to this genotype, such as neonatal intensive care unit admissions.
**Meta-Analyses and Reviews**	Recent meta-analyses would likely confirm the significant association between the rs726344 G polymorphism and preterm birth, possibly integrating data from multiple studies to reinforce this finding.
**Potential Mechanisms**	Hypothetical updates could involve the exploration of the biological mechanisms by which FNDC5 influences preterm labor, possibly through inflammatory pathways or effects on uterine contractility.
**Future Research Directions**	Suggestions for future research might include larger, multi-center studies to confirm these findings across different ethnic groups, and investigations into potential interventions to mitigate the risks associated with these genotypes.

Table S3. Correlation of Irisin with Gestational Diabetes Mellitus

Aspect	Description	References
**Gestational Diabetes Mellitus (GDM)**	GDM is a form of diabetes occurring during pregnancy characterized by high blood glucose levels, posing risks to both mother and developing baby. The underlying causes include hormonal changes that block insulin.	[115]
**Role of Irisin**	Irisin improves glucose metabolism and insulin sensitivity, indicating its potential in managing and preventing metabolic disorders like diabetes.	[117]

**Irisin Levels in GDM**	Women with GDM show significantly lower irisin levels compared to normal pregnancies. Postpartum, these differences are not significant.	[117]
**Irisin and Exercise**	Recent studies suggest that exercise may enhance irisin levels, potentially reducing the risk of developing GDM, especially in overweight and obese women.	[98]
**Prediction and Early Detection**	Lower irisin levels during the first trimester may help predict GDM, aiding in early intervention strategies.	[118, 120]
**Novel Biomarkers in GDM**	Other biomolecules like FABP4, osteocalcin, and adiponectin are being studied as potential predictors of GDM, alongside irisin.	[121]
**Future Research**	Further investigation is needed to fully understand irisin's mechanisms in GDM prevention, its role as a predictive biomarker, and its therapeutic potential.	[119, 121]

Table S4. Key points of Irisin role in PCOS

Reference	Key Points
**[122]**	PCOS is characterized by polycystic ovaries, anovulation, and hyperandrogenism.
**[123]**	Metabolic disorder is a critical factor in the occurrence of PCOS.
**[124, 125]**	Researchers have found a significant association between irisin levels and PCOS.
**[24]**	Irisin concentration is significantly higher in PCOS patients compared to controls.
**[24]**	Positive correlation between irisin concentration and severity of insulin resistance.
**[128]**	Irisin involved in the pathophysiology of PCOS; BAT role in metabolic balance via heat production.
**[130]**	Significant decrease in BAT activity in PCOS patients.
**[127]**	Irisin activates BAT function in PCOS mice, improving insulin sensitivity and reducing ovarian cysts.
**[23]**	Metformin treatment in PCOS patients significantly reduced increased irisin levels.
**[131]**	Irisin reduces insulin resistance and improves insulin sensitivity, aiding glucose management in PCOS.
**[132]**	Obesity is a risk factor for PCOS; weight management is crucial in treatment.
**[133]**	Irisin may help in body fat reduction and weight loss.
